# Assessment of Physical Fitness in Children and Adolescents with Simple Obesity

**DOI:** 10.3390/children12101388

**Published:** 2025-10-15

**Authors:** Jacek Podogrodzki, Mieczysław Szalecki, Anna Wrona, Aldona Wierzbicka-Rucińska

**Affiliations:** 1 Department of Neurology and Epileptology, The Children’s Memorial Health Institute, Aleja Dzieci Polskich 20, 04-730 Warsaw, Poland; jacekpodogrodzki@wp.pl; 2 Clinic of Endocrinology and Diabetology, The Children’s Memorial Health Institute, Aleja Dzieci Polskich 20, 04-730 Warsaw, Poland; m.szalecki@ipczd.pl; 3 Department of Science, The Children’s Memorial Health Institute, Aleja Dzieci Polskich 20, 04-730 Warsaw, Poland; anna.wrona@vp.pl; 4 Department of Clinical Biochemistry, The Children’s Memorial Health Institute, Aleja Dzieci Polskich 20, 04-730 Warsaw, Poland

**Keywords:** physical fitness, EUROFIT test, children and adolescents, simple obesity

## Abstract

**Highlights:**

**What are the main findings?**

• Children with obesity showed a multidimensional profile of health and fitness: balance, speed, functional strength, and agility were significantly impaired, while flexibility remained within the normal range and handgrip strength was above reference values.
• Sex-related differences were observed: girls outperformed boys in half of the fitness tests, indicating gender-specific patterns in physical performance.

**What is the implication of the main finding?**

• Identification of specific strengths and weaknesses in physical fitness can guide the development of tailored rehabilitation and physical activity programs for children with obesity. Considering gender differences is crucial when designing personalized interventions to optimize effectiveness and engagement in pediatric obesity management. These results may have practical implications for designing personalized interventions aimed at improving physical fitness in children and adolescents with obesity.
• The observed sex-specific patterns of motor performance in children with simple obesity suggest that biological maturation and the endocrine profile may modulate the impact of excess body weight on physical fitness. These findings highlight the need to consider developmental and hormonal factors when interpreting physical performance outcomes in pediatric populations with obesity.

**Abstract:**

Objectives: The systematic increase in the number of overweight and obese people in recent years has led to the recognition of this condition as a chronic, non-infectious disease of civilization, declared a global epidemic by WHO in 1997. This phenomenon is particularly dangerous in children, because it negatively affects their later existence in the health, mental and social spheres. This phenomenon is particularly concerning in the pediatric population, as it may have long-term adverse effects on physical health, psychological well-being, and social functioning. Objective: The aim of this study was to assess anthropometric parameters and physical fitness using the EUROFIT test in children and adolescents diagnosed with obesity. Materials and Methods: The study group consisted of 123 pediatric patients attending the Endocrinology and Diabetology Clinic and Pediatric Rehabilitation IP-CZD aged 8–16 (64 boys—52% and 59 girls—48%) with diagnosed simple obesity. Obesity was diagnosed according to the CDC standard using percentile charts from the OLAF study. Physical fitness was assessed using the EUROFIT test using 8 samples, and body mass composition was examined using the bioimpedance method with the BC 418 Tanita analyzer. Results: The results of our own research obtained in this study were compared to population standards. The total results of the EUROFIT test in the study group were statistically significantly lower than the norm. The results of the balance, upper limb movement speed, jumping, trunk strength, functional strength and agility tests were lower than the norm, the flexibility result was within the norm, and only hand strength was higher than the norm. In 4 out of 8 fitness tests, girls achieved significantly better results than boys. Conclusions: Reduced physical fitness is characteristic of children and adolescents with simple obesity. Worse physical fitness shows significant correlations with the results of anthropometric measurements.

## 1. Introduction

In recent years, the global rise in overweight and obesity has led to their recognition as chronic non-communicable diseases, often referred to as civilization diseases [1]. In 1997, the World Health Organization (WHO) declared obesity a global epidemic, and its prevalence has continued to increase, particularly among children and adolescents [2,3]. This trend is alarming, as early-onset obesity has long-term consequences for physical health, mental well-being, and social functioning [4]. Simple obesity, also referred to as primary obesity, is a non-syndromic form of obesity that results primarily from a chronic positive energy balance where energy intake consistently exceeds energy expenditure. It is the most prevalent type of obesity and is not associated with identifiable secondary causes such as endocrine disorders, genetic syndromes, or medication use. Simple obesity typically develops due to a combination of behavioral, environmental, and lifestyle factors, including excessive caloric intake, sedentary behavior, and inadequate physical activity. Obesity-related complications may manifest during childhood or emerge later in life, but in both cases, they tend to occur prematurely [5]. Psychosocial consequences often accompany the onset of obesity, including low self-esteem, depressive symptoms, and peer discrimination [6,7,8,9,10]. These factors, considered environmental, play an important role in the pathogenesis of obesity [11]. In modern societies, declining physical activity is strongly linked to technological advancements and the widespread availability of labor-saving devices [12,13]. Numerous studies have demonstrated a close association between increased physical activity and weight reduction, as well as an inverse relationship between body mass index (BMI) and physical activity levels [14,15,16]. The aim of the present study was to assess the physical fitness of children and adolescents aged 8–16 years with simple obesity and to analyze the associations between physical fitness and selected anthropometric parameters in this population. The novelty of this study lies in its comprehensive approach, combining anthropometric assessment, body composition analysis using bioimpedance, and a full evaluation of physical fitness with the EUROFIT test in children with simple obesity. Unlike many previous studies that focused on single parameters, our work provides a multidimensional profile of health and fitness in this population. Importantly, the diagnosis of obesity was based on Polish reference charts (OLAF), which increases the relevance of the findings for the local pediatric population. Moreover, the study revealed that not all motor abilities are equally impaired in obese children: while balance, speed, functional strength, and agility were significantly reduced, flexibility remained within the normal range and handgrip strength was even above the reference values. The identification of such specific patterns of strengths and weaknesses represents an original contribution. In addition, we demonstrated sex-related differences, with girls outperforming boys in half of the fitness tests, which highlights the need to consider gender when planning rehabilitation and physical activity programs.

## 2. Materials and Methods

The study was cross-sectional and enrolled 123 patients aged 8–16 years from the Department of Endocrinology and Diabetology and the Department of Pediatric Rehabilitation at The Children’s Memorial Health Institute (IP-CZD) in Warsaw, Poland. The cohort included 64 boys (52%, mean age: 12.63 years) and 59 girls (48%, mean age: 12.68 years), all diagnosed with simple obesity.

Body mass index (BMI) percentiles specific to Polish children and adolescents, as published in the OLAF study, were used for assessment. Obesity was defined according to CDC criteria as a BMI ≥ the 95th percentile for age and sex. Exclusion criteria included the presence of chronic endocrine, metabolic, genetic, neurological, or gastrointestinal disorders, as well as any other condition potentially contributing to secondary obesity. Patients diagnosed with obesity-related complications—such as non-alcoholic fatty liver disease, hypertension, insulin resistance, or type 2 diabetes—were also excluded. Children younger than 8 years or older than 16 years, as well as those for whom informed consent was not obtained from either the patient or legal guardians, were not included in the study. The study protocol was approved by the Bioethics Committee of the Children’s Memorial Health Institute (Resolution No. 71/KBE/2013).

### 2.1. EUROFIT Physical Fitness Assessment

Physical fitness was assessed using the EUROFIT test battery, a standardized protocol developed by the Council of Europe to evaluate multiple components of physical fitness. The battery included the following tests: Flamingo Balance Test (static balance), Plate Tapping (upper limb speed and coordination), Sit-and-Reach (flexibility), Standing Broad Jump (explosive leg power), Handgrip Strength (maximal isometric strength), Sit-Ups in 30 s (abdominal muscle endurance), Bent Arm Hang (upper body muscular endurance), 10 × 5 m Shuttle Run (speed and agility), and the 20 m Endurance Shuttle Run (cardiorespiratory endurance). All tests were performed according to the EUROFIT manual under standardized conditions by trained examiners. All tests were performed according to the EUROFIT manual under standardized conditions by trained examiners. In this study, eight of the nine tests were used. The endurance component (20 m shuttle run) was excluded due to patient safety considerations and the inability to perform the running test in a hospital setting.

Test results were expressed on a scale from 0 to 100 points. The scores were referenced against normative population data published by the Academy of Physical Education (AWF) in Poland.

### 2.2. Anthropometric Measurements

Anthropometric assessments were conducted in the morning, with participants fasting and dressed in underwear. Body height was measured using a Harpenden stadiometer, and body weight was assessed with a medical-grade scale. Waist circumference was measured using a non-elastic measuring tape. Body mass index (BMI) and Cole’s index were calculated. Body composition, including the percentage of fat mass (%BF) and fat-free mass (%FFM), was evaluated using bioelectrical impedance analysis with the Tanita BC-418 analyzer.

### 2.3. Statistical Analysis

To assess the normality of the distribution of variables, the Lilliefors and Shapiro–Wilk tests were used. Differences between the means of two independent groups were analyzed using Student’s *t*-test (for normally distributed variables). For variables not following a normal distribution, the Mann–Whitney U test was applied. For comparisons among multiple groups, one-way ANOVA was used for normally distributed variables, while the Kruskal–Wallis test was used for variables with non-normal distribution.

To examine the relationship between two continuous variables (with at least one normally distributed), linear regression and Pearson’s correlation coefficient (r) were used. For non-normally distributed variables, Spearman’s rank correlation was applied. Multivariate linear regression was employed to investigate relationships among three or more normally distributed continuous variables. For non-normally distributed continuous variables, linear transformation was performed prior to analysis. Linear discriminant analysis was also applied to determine the contribution of individual variables in distinguishing between two groups, and a discriminant function was computed. Logistic regression was used to examine the effect of multiple independent variables (with no distribution assumptions) on a dichotomous dependent variable (e.g., sex). To compare the frequency of a given characteristic between two groups, the structural index test was used. Statistical significance was set at *p* < 0.05.

## 3. Results

The EUROFIT physical fitness test results for the study group are summarized in Table 1. The EUROFIT test shows the mean overall physical fitness scores, calculated as the sum of eight test components. The average score for the entire cohort was 33.48 points, which is significantly lower than the normative reference value of 50 points. This suggests that, on average, the children included in the study demonstrated reduced physical fitness compared to standardized population norms. When analyzed by gender, girls achieved a higher mean score (34.95 points) than boys (32.13 points). Despite this difference, both groups scored significantly below the normative value (*p* < 0.05), indicating a general decrease in physical fitness across all tested components. Statistical analysis confirmed that the deviations from population norms were highly significant for the overall group (N = 123, *p* = 0.00001), as well as separately for girls (N = 59, *p* = 0.00001) and boys (N = 64, *p* = 0.00001). Both groups performed significantly below the normative standard (*p* < 0.05), indicating a reduced level of physical fitness across all eight tested components.

Table 1 presents the results of individual components of the EUROFIT test for the study group (N = 123). The analysis revealed that performance in several areas was significantly below the normative standard of 50 points. Specifically, balance (27.81 points), upper limb movement speed (43.79 points), jumping ability (31.81 points), trunk strength (26.67 points), functional strength (1.21 points), and agility (32.80 points) were all significantly lower than the population norm (*p* < 0.05). In contrast, hand strength was the only component where participants scored significantly above the norm, with an average of 54.58 points (*p* < 0.05). The only component where the study group did not differ significantly from the normative value was flexibility, with a mean score of 49.12 points (*p* = 0.38), indicating performance in line with population expectations. These findings suggest that while some areas of physical fitness remain relatively preserved (e.g., hand strength and flexibility), the majority of tested components demonstrate notable deficiencies in the study population. In both sexes, scores were significantly lower than population norms (*p* < 0.05) for the majority of test items, particularly those requiring dynamic effort and movement of body mass against gravity, such as the standing broad jump (Jumping Ability), agility, and trunk strength. Flexibility was the only component in which girls achieved scores comparable to the population norm, while boys’ flexibility scores were significantly lower. Girls achieved significantly higher mean scores than boys in flexibility, jumping ability, and agility. The most pronounced performance deficits in both sexes were observed in functional strength, where average scores were substantially below the norm, and in balance, where results showed wide variability, with some participants scoring zero points. Handgrip strength was also lower than normative values in both sexes, despite relatively better results in girls. These findings highlight that in children and adolescents with obesity, the greatest impairments occur in physical fitness components requiring the movement of body weight and postural control, while flexibility is least affected, particularly in girls.

**Figure 1:**
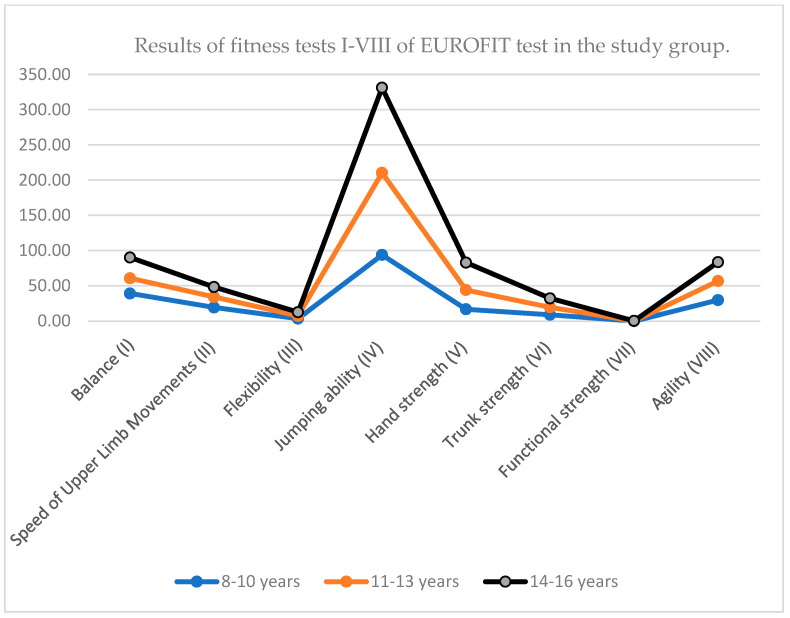
Results of fitness tests I–VIII of EUROFIT test in the study group.

Overall, the data indicate that muscular strength, trunk stability, flexibility, and jumping ability improve steadily with age, while balance, agility, and speed of upper limb movements show a decline, particularly during early adolescence. These trends likely reflect interactions between growth, body composition changes, and neuromuscular development during puberty (Figure 1).

The data in the table reflect the typical development of motor skills in girls aged 8 to 16. Most motor abilities (strength, speed, balance) improve with age. However, numerous inconsistencies are evident in the table (such as data misalignments), particularly in the 14–16 age group, which hinders accurate interpretation. The data indicate a significant increase in motor performance during the 11–13 age period, which coincides with the phase of intensive physical maturation.

Table 2 compares the body mass composition parameters of girls (n = 59) and boys (n = 64) in the study group with population norms. The analysis revealed statistically significant differences between both sexes and the normative values across several components. Girls had an average fat mass percentage of 38.22%, while boys averaged 35.17%. Both values were significantly higher than the norm (*p* < 0.05), and the difference between girls and boys was also statistically significant (*p* = 0.012), indicating higher fat accumulation in girls. Regarding absolute fat mass (kg), girls averaged 29.72 kg and boys 28.48 kg, both significantly above normative values (*p* < 0.05). However, the gender difference in fat mass in kilograms was not statistically significant. In contrast, boys had significantly higher levels of fat-free mass (51.65 kg) compared to girls (46.65 kg) (*p* = 0.035), and both groups exceeded normative expectations (*p* < 0.05). This suggests greater lean body mass in boys. A similar pattern was observed for total body water content, where boys (37.68 kg) scored higher than girls (34.61 kg), but the gender difference was not statistically significant, despite both values being significantly higher than the norm (*p* < 0.05). Overall, the data indicate that both fat mass and lean tissue parameters (fat-free mass and water content) were elevated in the study group, with some significant gender-specific differences in body composition.

This Table 3 presents a comparison of somatic characteristics between girls (n = 59) and boys (n = 64) in the study group, based on both calendar age and growth age. Despite having a similar calendar age (mean = 12.6 ± 0.3 years), the growth age was higher in girls (14.4 ± 0.4 years) than in boys (13.5 ± 0.3 years), indicating that girls were generally at a more advanced stage of biological development. Girls had a Z-score of 0.97 for body height, and boys had a Z-score of 0.64, both significantly higher than the population norm (*p* < 0.05), suggesting above-average stature in both groups. However, the difference between girls and boys was not statistically significant (NS). Body weight Z-scores were significantly elevated in both girls (4.13) and boys (3.32) compared to the norm (*p* < 0.05). Similarly, based on growth age, both groups still showed significantly higher weight than the standard, with slightly lower but still elevated Z-scores. The gender difference was statistically significant (*p* < 0.05), indicating that girls tended to have higher relative body weight than boys. Waist circumference was also significantly above the normative value in both sexes (Z-scores: girls = 6.12, boys = 4.91, *p* < 0.05). These results suggest central adiposity in both groups, more pronounced in girls. BMI values were elevated in both girls (Z = 4.12) and boys (Z = 4.16) relative to norms (*p* < 0.05). No statistically significant differences were observed between sexes in BMI scores (NS), suggesting similar trends in general overweight/obesity across both groups. The study group showed significantly elevated somatic indicators (height, weight, waist circumference, and BMI) compared to normative data. Girls exhibited slightly more advanced biological maturity and higher values in several parameters, though not all differences between sexes reached statistical significance (Table 4).

The present analysis demonstrates that both the age of onset and the duration of obesity are significantly related to anthropometric and body composition parameters in children. An earlier onset of obesity was associated with higher deviations from reference BMI values, as reflected by negative correlations with BMI SD and Cole’s index. In contrast, the duration of obesity showed stronger positive associations with body weight, BMI, fat-free mass, and total body water, indicating that prolonged obesity is linked to progressive increases in these parameters (Table 4, Table 5 and Table 6). Breastfeeding, although generally weakly correlated with the studied outcomes, revealed a modest protective trend, particularly against higher fat percentage. These findings highlight the importance of early prevention strategies, as both the timing and chronicity of obesity contribute to adverse body composition profiles in childhood.

## 4. Discussion

**Figure 2:**
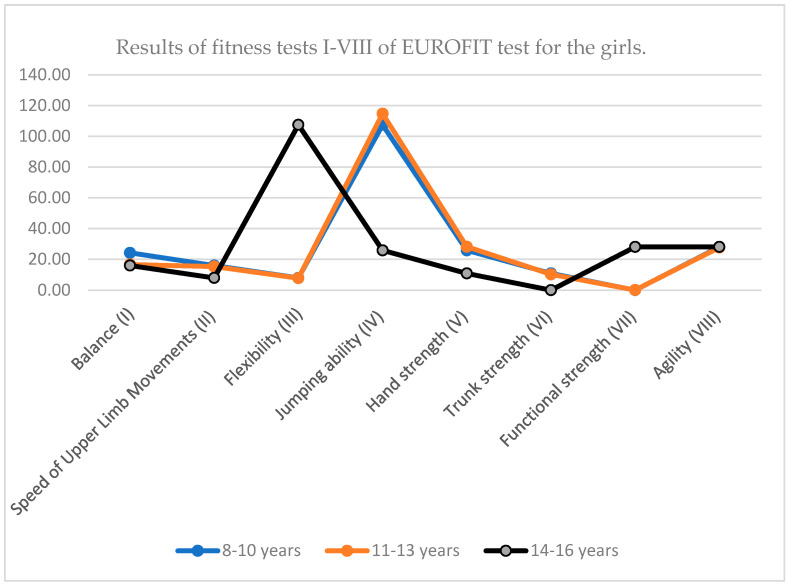
Results of fitness tests I–VIII of EUROFIT test for the girls.

**Figure 3:**
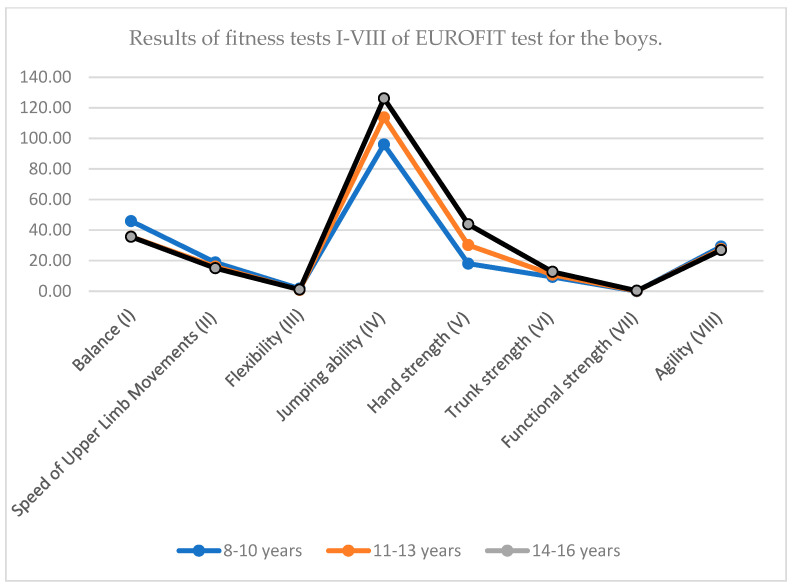
Results of fitness tests I–VIII of EUROFIT test for the boys.

The EUROFIT test administered to children with simple obesity revealed a general reduction in performance compared with population norms. In the study group (N = 123), balance test results showed significant negative correlations with body weight, body weight Z-score, height Z-score, waist circumference, fat mass (both percentage and kilograms), BMI, BMI Z-score, and Cole’s index. Among boys, balance performance was inversely associated with all of these parameters, whereas in girls, significant correlations were observed for body weight Z-score, fat mass (% and kg), BMI, BMI Z-score, and Cole’s index. These findings suggest that excess body weight—particularly its fat component—and weight–height indices exert a detrimental effect on balance performance. In boys, additional negative associations with height Z-score and waist circumference may partly explain their slightly poorer outcomes compared with girls, although sex differences did not reach statistical significance. Similar results were reported by Barańska [17], Popławska et al. [18], Popławska and Dmitruk [19], and Fiori [20]. Also confirmed an inverse relationship between balance performance and BMI [21]. The balance test, performed in a standing position, required the activation of postural muscles to counteract gravity and stabilize body sway, especially in the frontal plane. Excess body weight increases the mechanical load on these muscles, which likely contributes to reduced test scores. In the upper limb movement speed test (N = 123), significant negative correlations were found with waist circumference, fat mass (% and kg), BMI, BMI Z-score, and Cole’s index (Table 6). In boys (N = 64), poorer results were linked to waist circumference, BMI, and BMI Z-score, while in girls (N = 59), significant associations were observed with fat mass (%) and Cole’s index. Interestingly, in girls, performance showed a positive correlation with height Z-score. This may reflect a mechanical advantage, as greater height and arm span can facilitate task execution. These sex-specific determinants—abdominal obesity more prominent in boys, and higher body fat percentage in girls—appear to influence test outcomes. Comparable findings have been reported by Popławska et al. [21] and Sobieska et al. [22]. Negative associations with body weight were also confirmed by Barańska [23] and Kocaj and Jabłoński [24]. Taken together, these results indicate that excess body mass, particularly abdominal fat and overall adiposity, is strongly linked to impaired balance and upper limb movement speed in children with obesity. The additional load on postural and shoulder girdle muscles likely underlies the observed deficits, highlighting the role of somatic composition in physical fitness outcomes. In our study (N = 123), overweight and obesity in children were associated with reduced physical fitness, particularly in tasks requiring the movement of body mass against gravity. Fat mass and waist circumference were the strongest negative predictors of performance across multiple tests, highlighting their key role in limiting dynamic and endurance-based abilities. Flexibility was the only motor ability whose scores did not differ significantly from population norms (Table 2). In girls, no significant correlations with anthropometric measures were observed, suggesting that sex-related predispositions may mitigate the effects of excess body weight. In boys, negative correlations were found with body weight, weight Z-score, height, waist circumference, and fat mass (kg). The static nature of the test likely explains why subcutaneous fat, even in mild obesity, does not impair performance, and a significant association between flexibility and sex was confirmed [23,25,26]. Jumping performance was negatively correlated with multiple anthropometric measures in the total sample and in boys, including age, body weight, weight Z-score, height, waist circumference, fat mass (% and kg), lean body mass, water content, BMI, BMI Z-score, and Cole’s index. In girls, correlations were observed with body weight, weight Z-score, waist circumference, fat mass (% and kg), BMI, BMI Z-score, and Cole’s index (Figure 2). Girls achieved significantly higher scores, consistent with previous studies [23,24,27,28,29]. Lower performance in children with obesity likely reflects the need to move greater body mass against gravity. Handgrip strength correlated positively with body weight Z-score, height, height Z-score, lean body mass, and water content. In boys, fat mass (%) was negatively associated, while in girls, no adverse effect of fat mass was observed. These findings indicate that body weight and height, as markers of biological development, are key determinants of handgrip strength [24,30,31,32,33,34,35]. Trunk strength was negatively associated with body weight, weight Z-score, waist circumference, fat mass (% and kg), BMI, BMI Z-score, and Cole’s index. In girls, correlations were observed with waist circumference, fat mass, BMI, and Cole’s index, while boys exhibited negative associations with a broader range of anthropometric variables. Excess waist circumference and fat mass likely impair performance due to mechanical and endurance-related factors [24,27,28,33]. Assessment of functional strength was limited, particularly among girls (only one participant completed the test). In boys, functional strength correlated negatively with fat mass (%), as additional weight represents an extra load in a test requiring supporting one’s own body weight [24,27,30,34,36]. Agility performance showed negative correlations with anthropometric measures in the total sample and in both sexes, including age, body weight, weight Z-score, height, waist circumference, fat mass (% and kg), lean body mass, water content, BMI, BMI Z-score, and Cole’s index (Figure 3). Reduced agility in children with obesity likely reflects the frequent directional changes required and the impact of excess body mass. Girls achieved significantly better results than boys in half of the EUROFIT tests, reflecting sex-related differences in anthropometry and biological development [18,19,27,34,37,38]. Overall, overweight and obesity are associated with marked reductions in physical fitness, particularly in dynamic and endurance-based tasks. Flexibility was the least affected component, with results comparable to population norms, especially in girls. Fat mass and waist circumference consistently showed the strongest negative correlations with performance, confirming the EUROFIT battery as a valuable tool for identifying motor abilities most compromised by excess body weight in children and adolescents. It should also be emphasized that biological maturation and the associated hormonal milieu represent important determinants of motor performance during childhood and adolescence. Testosterone, which increases markedly in boys during puberty, promotes the development of muscle mass, strength, and anaerobic capacity, while estrogens influence body composition, fat distribution, and flexibility, particularly in girls. These endocrine factors interact with somatic growth and may partly explain the sex-related differences observed in physical fitness outcomes. However, in the present study, no blood samples were collected, and therefore circulating hormone levels could not be assessed. This methodological limitation precluded direct analysis of the associations between sex steroids and motor abilities. It should be emphasized that biological maturation and the associated hormonal milieu represent important determinants of motor performance during childhood and adolescence. Testosterone, which rises markedly in boys during puberty, promotes increases in muscle mass, strength, and anaerobic performance, whereas estrogens contribute to the development of body composition, fat distribution, and flexibility, particularly in girls. These endocrine factors interact with somatic growth and may partly explain sex-related differences observed in physical fitness outcomes. However, in the present study, no blood samples were collected, and therefore circulating hormone levels could not be assessed. This methodological limitation precluded direct analysis of the associations between sex steroids and motor abilities. Future research should incorporate biochemical assessment of hormonal status alongside anthropometric and fitness testing, as this would provide a more comprehensive understanding of the biological mechanisms underlying reduced physical performance in children with obesity [39]. Future research should incorporate biochemical assessment of hormonal status alongside anthropometric and fitness testing, as this would provide a more comprehensive understanding of the biological mechanisms underlying reduced physical performance in children with obesity.

The results indicate that while most strength-related variables (e.g., hand strength, trunk strength, jumping ability) improve with age, some abilities such as balance and agility may plateau or even decline slightly during adolescence. These findings highlight the importance of age-specific training and monitoring in youth physical development programs.

## 5. Conclusions

Simple obesity in children and adolescents is associated with a marked reduction in physical fitness, particularly in tasks that require moving body mass against gravity, such as standing broad jump, agility, and trunk strength. Flexibility was the least affected component, with results comparable to population norms, especially among girls, likely due to sex-related biological predispositions. Fat mass and waist circumference showed the strongest negative correlations with fitness scores, confirming their role as major limiting factors for dynamic and endurance-based performance. The EUROFIT test battery proved useful in identifying which motor abilities are most impaired in children and adolescents with overweight or obesity. In addition, the age of onset and duration of obesity were significantly related to anthropometric and body composition parameters. Earlier onset of obesity was linked to higher relative BMI values, whereas longer duration was associated with progressive increases in body weight, BMI, fat-free mass, and total body water. Breastfeeding showed only weak associations with body composition, though a modest protective trend against higher fat percentage was observed. These findings emphasize the importance of early prevention strategies, since both the timing and chronicity of obesity negatively affect physical fitness and body composition in childhood.

### Limitations of the Study

Single-center design: The study was conducted in one clinical center, which may limit the generalizability of the results to the wider pediatric population. Relatively small sample size—although 123 participants provide valuable insights, a larger cohort would strengthen the statistical power and allow for more detailed subgroup analyses. Cross-sectional nature: The study design does not allow for assessment of causality or long-term changes in physical fitness and body composition over time. Lack of control group: Comparison was made only to population norms, without including a contemporaneous control group of healthy children assessed with the same methodology. Potential measurement bias: The EUROFIT test, while standardized, may be influenced by children’s motivation, learning effect, or examiner variability. Limited scope of clinical data: The study focused on anthropometric and fitness parameters, but did not include biochemical or hormonal markers that could further characterize the health risks associated with obesity. Age and puberty variability: The wide age range (8–16 years) may introduce heterogeneity related to pubertal development, which was not fully controlled for. In the cohort of children with obesity, the assessment protocol was limited to standardized physical fitness testing. No venous blood sampling or biochemical analyses were performed within this study. Consequently, circulating concentrations of sex hormones such as testosterone and estrogens were not available. This methodological constraint precluded the possibility of examining associations between endocrine parameters and motor performance outcomes in the investigated groups.

## Figures and Tables

**Table 1:** Results of fitness tests I–VIII of EUROFIT test in the study group.

EUROFIT Test Items	Points Average	Min	Max	SD	*p*
Balance (I)	27.81	0	61	±16.32	*p* < 0.05
Speed of Upper Limb Movements (II)	43.79	8	69	±9.74	*p* < 0.05
Flexibility (III)	49.12	24	72	±10.23	NS
Jumping ability (IV)	31.81	3	53	±10.24	*p* < 0.05
Hand strength (V)	54.58	35	88	±9.24	*p* < 0.05
Trunk strength (VI)	26.67	1	56	±13.05	*p* < 0.05
Functional strength (VII)	1.21	0	41	±6.67	*p* < 0.05
Agility (VIII)	32.8	1	50	±10.17	*p* < 0.05

Statistically significant differences refer to deviations from the norm *p* < 0.05, the normative reference value of 50 points.

**Table 2:** Comparison of body mass composition scores of girls and boys in the study group with the norm.

	Girls		Boys		*p* (g/b)
	Average g = 59 (Min–Max)	SD	Average b = 64 (Min–Max)	SD	
Fat mass [%]	38.22 (28.0–71.3)	±6.58	35.17 (26.0–61.2)	±6.75	*p* < 0.05
Fat mass [kg]	29.72 (11.0–72.7)	±10.89	28.48 (13.0–68.6)	±11.60	NS
Fat-free mass [kg]	46.65 (28.4–67.0)	±10.48	51.65 (29.9–78.6)	±14.89	*p* < 0.05
Water content [kg]	34.61 (20.8–49.0)	±7.69	37.68 (21.9–57-5)	±10.86	NS

Statistically significant differences refer to deviations from the gender *p* < 0.05, g—girls, b—boys.

**Table 3:** Comparison with the norm of somatic characteristics regarding the nutritional status of girls and boys in the study group.

Somatic Characteristics	Girls		Boys		g/b
	Calendar Age 12.6 ± 0.3 Years	Biological Age 14.4 ± 0.4 Lat	Calendar Age 12.6 ± 0.3 Years	Biological Age 13.5 ± 0.3 Lat	Calendar/Biological Age
	Z-Score	Z-Score	Z-Score	Z-Score	*p*
Body height [cm]	0.97 ± 0.15	0	0.64 ± 0.11	0	NS
Body weight [kg]	4.13 ± 0.22	3.71 ± 0.21	3.32 ± 0.15	2.90 ± 0.15	*p* < 0.05
Waist circumference [cm]	6.12 ± 0.25	5.95 ± 0.25	4.91 ± 0.15	4.91 ± 0.15	*p* < 0.05
BMI	4.12 ± 0.63	3.91 ± 0.21	4.16 ± 0.35	3.98 ± 0.11	NS

Statistically significant differences refer to deviations from the norm *p* < 0.05, g—girls, b—boys.

**Table 4:** Correlations between the results of fitness tests I–VIII and the clinical data of the girls from the study group.

Girl’s	Balance I	Speed of Upper Limb Movements II	Flexibility III	Jumping Ability IV	Hand Strength V	Trunk Strength VI	Functional Strength VII	Agility VIII
Age of onset of obesity	0.01	0.07	−0.31	−0.24	−0.06	−0.08	0.00	−0.14
Duration of obesity	−0.01	−0.05	0.28	0.03	0.17	0.04	0.03	0.05
Breastfeeding	−0.17	0.17	0.08	0.04	0.26	0.08	−0.05	0.08

Among girls, there was a negative correlation between flexibility and age at onset of obesity, and a positive correlation with duration of obesity.

**Table 5:** Correlations between the results of fitness tests I-VIII and clinical data among boys in the study group.

Boy’s	Balance I	Speed of Upper Limb Movements II	Flexibility III	Jumping Ability IV	Hand Strength V	Trunk Strength VI	Functional Strength VII	Agility VIII
Age of onset of obesity	0.33	0.12	−0.05	0.06	0.01	0.15	0.14	0.17
Duration of obesity	−0.33	−0.29	−0.10	−0.36	−0.04	−0.29	−0.04	−0.41
Breastfeeding	−0.04	−0.07	0.06	0.14	−0.11	0.12	0.01	−0.03

Among boys, there was a positive correlation between the age of onset of obesity and balance, and a negative correlation between the duration of obesity and motor tests I, II, IV, VI, VIII.

**Table 6:** Correlations between the results of anthropometric measurements and clinical features in the study group.

Children	Age of Onset of Obesity WPO	Duration of Obesity CTO	Breastfeeding KP
Age [years]	0.35	0.37	0.01
Weight [kg]	0.21	0.39	0.03
Weight SD	−0.08	0.18	0.02
Height [cm]	0.28	0.34	0.09
Height SD	−0.12	−0.05	0.14
Abdominal circumference	0.18	0.28	0.00
Fat %	−0.07	0.07	−0.15
% Fat-free	0.10	0.30	−0.06
Fat-free	0.24	0.36	0.10
Water content (TBW)	0.23	0.35	0.09
BMI	0.03	0.32	0.02
SD_BMI	−0.10	0.18	0.01
Cole’s index	−0.15	0.03	−0.07

## Data Availability

The data supporting the findings of this study are available from the corresponding author upon reasonable request. Access is restricted due to privacy considerations and the presence of untranslated Polish entries related to the author’s doctoral research.

## Abbreviations

The following abbreviations are used in this manuscript:

AWF	Academy of Physical Education
BMI	Body Mass Index
CDC	Centers for Disease Control
HR-F	Health Related Fitness
HRQoL	Health-Related Quality of Life
IDF	International Diabetes Federation
IPCZD	The Children’s Memorial Health Institute
KBE	The local Bioethical Committee
SD	Standard Deviation
TBW	Total Body Water
WC	Waist Circumference
WHO	World Health Organization
WHR	Waist-to-Hip Ratio
WHtR	Waist-to-Height Ratio/Waist Circumference-to-Body Height Ratio
